# Evaluation of family planning and abortion education in preclinical curriculum at a large midwestern medical school

**DOI:** 10.1016/j.heliyon.2022.e09894

**Published:** 2022-07-09

**Authors:** Lucy Brown, Sarah Swiezy, Alexandra McKinzie, Sarah Komanapalli, Caitlin Bernard

**Affiliations:** Indiana University School of Medicine, 340 W 10th St, Indianapolis, IN 46202, USA

**Keywords:** Abortion, Medical education, Undergraduate medical curriculum, Pregnancy options counseling, Contraceptive counseling

## Abstract

**Objective:**

Evaluate a Midwestern medical school's current pregnancy termination and family planning undergraduate medical curriculum (UMC) in accordance with Association of Professors of Gynecology and Obstetrics (APGO) guidelines. Assess 1) student interest 2) preparedness to counsel patients, and 3) preferred modality of instruction.

**Study design:**

A survey assessed students about UMC. Course syllabus learning objectives and APGO educational guidelines were compared.

**Results:**

There were 309 responses total; six did not complete all survey questions and were excluded. Participants (n = 303) were primarily female (62%) and White (74%). Across all class levels, many (61%) students expected to learn about family planning and contraception in UMC. While most (84–88%) participants who completed the preclinical course with or without the clerkship felt prepared to counsel about common, non-controversial pharmacotherapies, only 20% of students felt prepared to counsel on abortion options, and 75% of students who had completed both the preclinical and OBGYN clerkship felt unprepared for abortion counseling Overall, 86% of all students surveyed believed that the medical school should enhance its reproductive health coverage in UMC. Traditional lectures, panels, and direct clinical exposure were the most popular instructional modalities.

**Conclusion:**

We identified potential gaps in UMC where students expressed high level of interest with low level of preparedness regarding abortion options counseling, even among senior students. Considering the high percentage of students expecting to learn about pregnancy termination and family planning in their UMC, this expectation is not being met. Students were open to a variety of modalities of instruction, indicating that several possible options exist for curricular integration.

**Implications:**

Despite evidence of need for training in family planning and abortion, few medical institutions have a standardized curriculum. Little available literature exists on curricula covering pregnancy options and contraception counseling, signifying a gap of knowledge and an opportunity to study how to integrate these important topics into UMC.

## 1 Introduction

Given that 25% of US women will seek an abortion before age 45, there is an urgent need to expand healthcare providers' foundational knowledge surrounding abortion and family planning [1]. Abortion clinics across the country are closing, leaving six states–three in the Midwest alone–with only one clinic remaining [2]. To improve access to full-spectrum reproductive health care in the Midwest, we need a physician workforce that is educated and able to provide these services.

Despite the fact that abortion care is part of the required medical education curriculum, among 126 accredited American medical schools, 17% reported no formal educational curriculum about abortion in the clinical or preclinical years in 2005 [3]. An effective way to address medical education gaps in women's reproductive healthcare is via integration of the Association of Professors of Gynecology and Obstetrics (APGO) contraception and abortion student learning objectives into the course learning objectives defined in syllabi of medical school curricula [4]. APGO has established that, by graduation, medical students should have fundamental knowledge and specific clinical skills related to contraception and abortion. While APGO medical education guidelines majorly assist in development of clinical clerkship curricula, it also serves as a standard for evaluation of preclinical skills, such as nondirective counseling and knowledge of the public health impact of the legal status of abortion. To achieve higher-order skills (such as “counseling”) by the end of medical school, the foundational topics, i.e. abortion and contraception, necessarily must be broached in the preclinical coursework. Just as medical schools would not wait until the family medicine clerkship to teach the mechanism of action and indications for beta blockers, medical schools should similarly not wait until the OBGYN clerkship to teach about abortion and contraception.

In addition to fostering development of physicians who are competent and empathetic in various clinical scenarios, comprehensive reproductive health knowledge is necessary for success on rotations and standardized residency entrance exams, including the National Board Medical Exams (NBMEs), the United States Medical Licensing Examination (USMLE), and the Comprehensive Osteopathic Medical Licensing Examination (COMLEX). Researchers at the University of Colorado School of Medicine surveyed third-year medical students regarding abortion knowledge [5]. They utilized undergraduate web-based interactive self-examination (uWISE) questions published by APGO to determine whether the learning objectives defined by APGO were being met. Among the 127 participating students, the average score on knowledge-based questions was 47%, translating to a failing NBME, USMLE, or COMLEX score in this subject area and indicating a need for improved reproductive health education in accordance with APGO guidelines.

Despite the evidence of need for training in these topics, few medical institutions have a standardized family planning and abortion preclinical curriculum that incorporates all the APGO learning objectives. There is little available literature on reproductive health curriculum that covers pregnancy options, abortion, and contraception counseling, signifying a knowledge gap in this field and an opportunity to study how to best integrate these important topics into undergraduate medical education.

## 2 Methods

To assess the alignment between the medical curriculum and APGO's educational guidelines, session learning objectives (SLO) from the preclinical, didactic course Endocrine, Reproductive, Musculoskeletal, Dermatologic Systems (ERMD) syllabus and individual lectures were compared to the relevant APGO objectives.

The authors applied for Indiana University (IU) Kuali Protocol (v05.25.2021) and received Institutional Review Board (IRB #2010133460) exemption from IU School of Medicine (IUSM) to conduct the survey portion of this study. A 23-question survey (see Appendix A) was generated using Qualtrics. The first question of the survey had participants read about the study and acknowledge that they understood the information and consented to the use of their responses to the survey as research. Inspiration for these questions was taken from the Curriculum Reform Interest Survey [7] created and made publicly accessible by Medical Students for Choice (MSFC), though many questions were modified, and a majority were original. This survey was disseminated to students of all levels via unofficial class GroupMe and Facebook groups and the student newsletter. Students were prompted to read and agree to an informed consent on the first screen of the survey before proceeding to the questions. All non-demographic, multiple choice questions on the survey were pre-set to require a response. One standardized question was included as an internal control for participant effort and response validity; all responses marking an incorrect answer were removed from our sample. At the end of the survey, participants were entered into a gift card drawing. Money for the gift cards was provided by the Student Activism Fund of the MSFC chapter at our institution.

## 3 Results

### 3.1 Comparison of SLO

APGO and SLO from the 2021 ERMD course were compared to determine the extent to which the medical school adheres to these prescribed national guidelines. The APGO topics are generally well-covered for family planning topics but not for pregnancy termination topics [Table 1].

**Table 1:** Comparison of APGO family planning and pregnancy termination curriculum guidelines and 2021 ERMD SLO.

Alignment between APGO and ERMD SLO	Present	Absent
***Family Planning APGO SLO***		
Describe the mechanism of action and effectiveness of contraceptive methods	X	
Counsel the patient regarding the benefits, risks and use for each contraceptive method, including emergency contraception	X	
Discuss barriers to effective contraceptive use and reduction of unintended pregnancy, and how health policy, advocacy and social and environmental factors impact family planning and population health		X
Describe methods of male and female surgical sterilization	X	
Explain the risks, benefits and patient safety implications of female surgical sterilization procedures	X	
***Pregnancy Termination APGO SLO***		
Provide nondirective counseling to patients surrounding pregnancy, including unintended pregnancy		X
List surgical and non-surgical methods of pregnancy termination	X	
Identify potential complications of pregnancy termination	X	
Describe the public health impact of the legal status of abortion and discuss how health policy and advocacy, as well as social and environmental factors, impact access to abortion		X

### 3.2 Interest: survey

**Figure 1:**
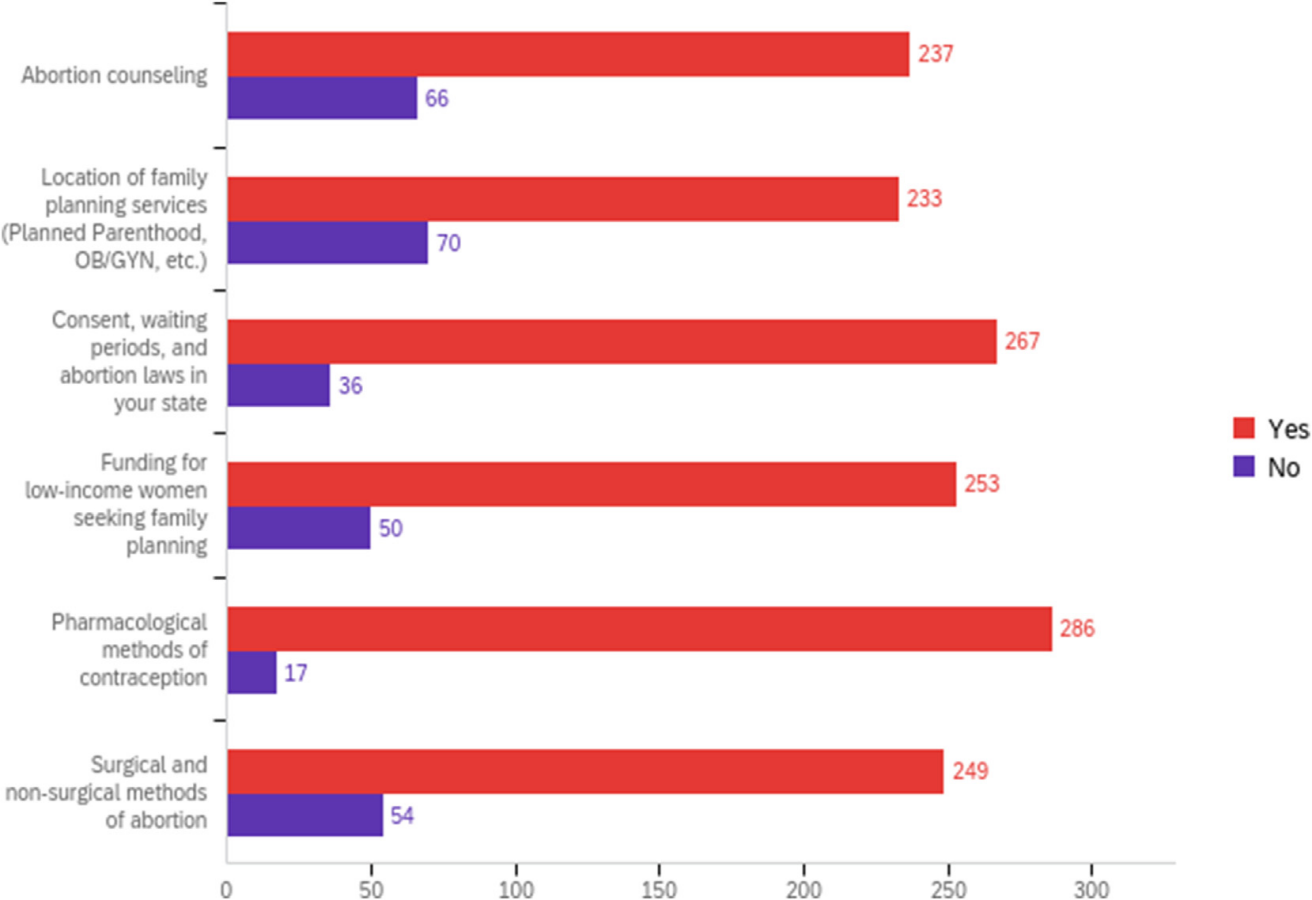
Participant interest in inclusion of specific reproductive health topics in Phase 1 curriculum.

Overall, 303 students completed the survey, representing 21% of the student body [Table 2]. There were 309 responses total; six did not complete all survey questions and were excluded. The remaining 303 responses were used in our analysis. To assess interest in reproductive health topics, all students were asked “Would you be interested in the following topics in Phase I (preclinical, didactic education) curriculum?” Overall, more than two-thirds of students demonstrated strong interest in every proposed reproductive health topic, including abortion counseling (78%), sites of available family planning services (77%), consent, waiting periods, and abortion laws in your state (88%), funding available for low-income women seeking family planning care (84%), pharmacological methods of contraception (94%), and surgical and non-surgical methods of abortion (82%) [Figure 1].

**Table 2:** Our study participants compared to available IUSM 2020–2021 demographic data [6].

Demographics	ERMD and OBGYN Clerkship (N = 108)	ERMD only (N = 76)	Neither (N = 119)	Total Study Participants (N = 303)	IUSM (N = 1441)	p value^b^
***Sex*, n (%)**						
Female	67 (62%)	49 (64%)	71 (60%)	187 (62%)	677 (47%)	**<0.0001**
Male	38 (35%)	25 (33%)	47 (40%)	110 (36%)	784 (53%)	
Non-binary	1 (1%)	0 (0%)	0 (0%)	1 (0.3%)		
Prefer not to say	2 (2%)	2 (3%)	1 (1%)	5 (2%)		
***Race***^a^**, n (%)**						
Caucasian	85 (77%)	60 (76%)	85 (71%)	230 (74%)	896 (62%)	**0.03**
Black or African American	1 (1%)	2 (3%)	7 (6%)	10 (3%)	94 (7%)	
Asian	16 (14%)	12 (15%)	21 (18%)	49 (16%)	223 (16%)	
Native Hawaiian/Other Pacific Islander	1 (0.9%)	0 (0%)	0 (0%)	1 (0.3%)	0 (0%)	
American Indian or Alaska Native	0 (0%)	0 (0%)	0 (0%)	0 (0%)	--	
Other	3 (3%)	3 (4%)	3 (3%)	10 (3%)	--	
***Ethnicity*, n (%)**						
Hispanic or Latino	5 (5%)	3 (4%)	8 (7%)	16 (5.3%)	166 (11.5%)	**0.003**
Nor Hispanic or Latino	96 (90%)	70 (92%)	106 (89%)	272 (90.1%)	1275 (88.5%)	
Prefer not to say	6 (6%)	3 (4%)	5 (4%)	14 (4.6%)		
***Home Geographic Region***, **n (%)**						
***In-State***					1140 (78%)	**0.05**
Midwest	93 (86%)	64 (85%)	95 (81%)	252 (83%)		
***Out-of-State***					320 (22%)	
Northeast	5 (5%)	4 (5%)	7 (6%)	16 (5%)		
Southeast	4 (4%)	3 (4%)	8 (7%)	14 (5%)		
Southwest	2 (2%)	2 (3%)	3 (3%)	6 (2%)		
West	3 (3%)	1 (1%)	2 (2%)	6 (2%)		
Other	1 (1%)	1 (1%)	3 (3%)	7 (2%)		
Missing^c^	0 (0%)	1 (1%)	1 (1%)	2 (0.7%)		

a Respondents were able to choose more than one response for “Race,” leading to 309 responses in this category.

b Chi-square statistic was used to compare categorical measures across groups.

c Not all respondents answered every question in the demographic portion of the survey; therefore, the totals for each group do not add to 303; we had 303 fully completed surveys. Those that did not answer a specific question are added in the “missing” rows for each category on the table.

To explore students' assessment of their didactic ERMD course's reproductive and sexual health content, all survey participants were asked to respond to the question, “Do you believe that IUSM should enhance its reproductive and sexual health coverage in the current curriculum, including expanding family planning and contraception didactic training?” 86% of students responded ​“Yes”.

### 3.3 Interest: free response

The written responses received by students who had completed both the ERMD and OBGYN clerkship (n = 108) were thoughtful and reflective. Overall, 105 students responded to this question. Although 12% (n = 13) responded with “N/A,'' “none,” or “nothing,” among other respondents, there was general discontent with the current curriculum and several suggestions were made. We found four major themes that best represented the dataset [Table 3].

**Table 3:** Free responses to, “What do you wish you had known about family planning and contraception before starting your OB/GYN Clerkship?”

Options	*“We had lectures in pharmacological contraception. But we get minimal discussion of abortion procedures including techniques and complications/follow-up instruction, and zero discussion of anything outside of pharmacologic or surgical family planning such as natural family planning.”* *“Options for low income and socioeconomic status individuals.”*
Navigating conversations with patients	*“More about abortion and overall techniques/strategies of how to communicate family planning and contraception topics with all patients, including pediatric and geriatric.”* *“I wish we'd practiced more with discussing options and doing full pro/con lists with patients. OBGYN attendings think the med students know all of the options, contraindications, pros/cons, etc.”* *“Seeing a doctor's "spiel" when walking through the options with a patient and having us practice that conversation would be helpful. A lot of the male students still seem so squeemish [sic] or have such akward [sic] phrasing of things. Get them practicing and seeing examples of good conversations [sic] about birth control options.”*
Abortion Policy	*“The specific Indiana laws regarding which forms of contraception are available the different time periods throughout pregnancy according to Indiana law. Also the knowledge of abortion providers to refer women to should they ask for that information.”* *“We learned birth control methods. No discussion on abortion care or those appropriate weeks of care. It would have been helpful to learn state law prior to the clerkship.”*
Miscarriage	*“Infertility issues, prevalence of miscarriages.”* *“How common miscarriages are and how to approach a patient who has had a miscarriage.”*

### 3.4 Preparedness

Only students indicating they had taken ERMD and/or the OBGYN clerkship (n = 159) were asked “Do you feel prepared to counsel patients on the following: abortion options, male contraception options, female contraception options, anti-depressants/anti-anxiety treatments and therapies, beta blockers, and diuretics?” We included beta blockers, antidepressants/anti-anxiety treatments and therapies, and diuretics to understand the level of self-confidence students have regarding reproductive health topics in comparison to topics that are non-controversial and thoroughly covered in the curriculum. Furthermore, the addition of topics outside of reproductive health allowed us to address potential confounding variables based on student variation in overall confidence regarding any topic.

**Figure 2:**
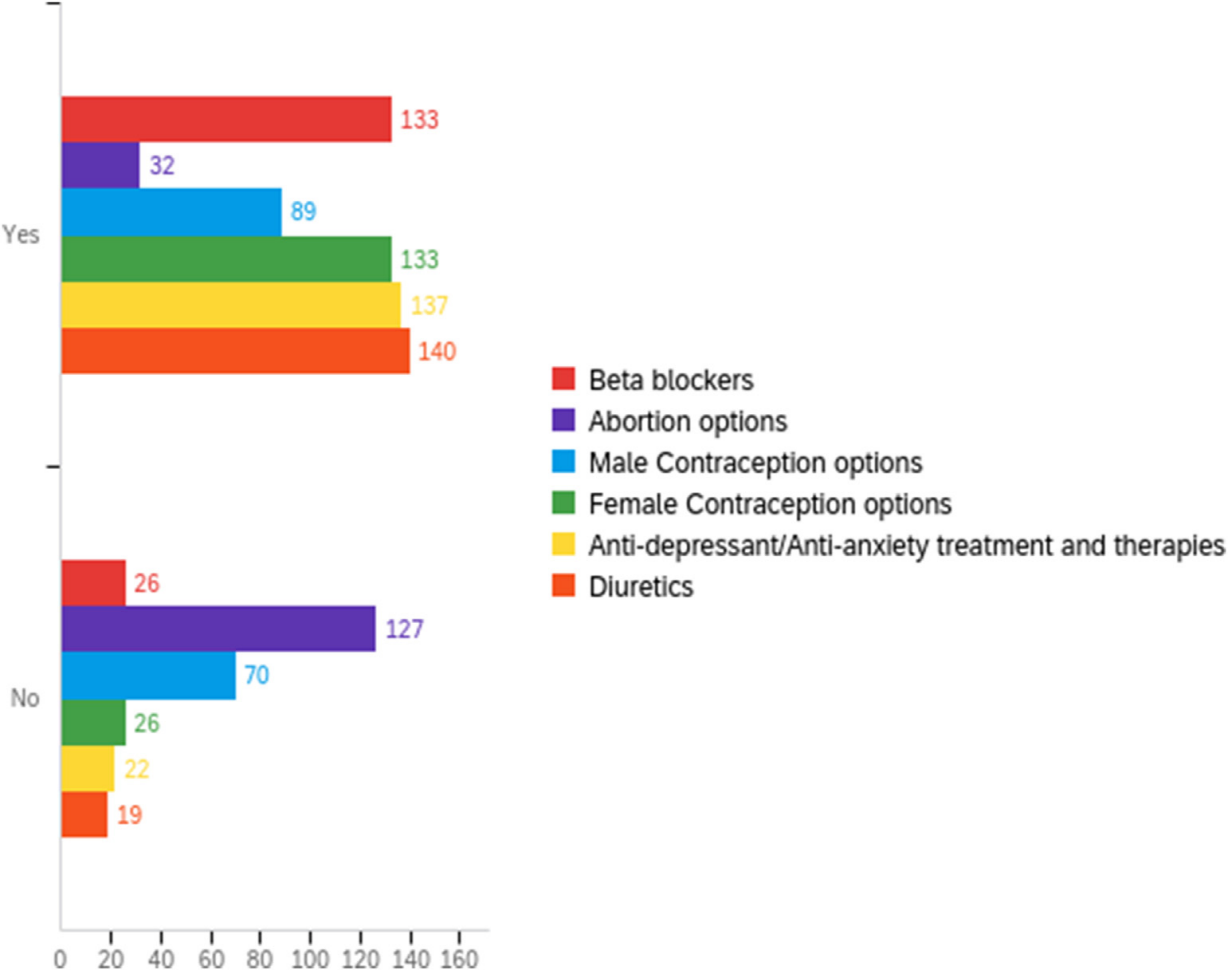
Participant responses to the question “Do you feel prepared to counsel patients on the following?” with comparison of topics in abortion/family planning to other non-controversial medical topics. 159 students answered this question.

While most participants reported feeling prepared to counsel patients about diuretics (88%), anti-depressants/anti-anxiety treatments and therapies (86%), and beta blockers (84%), only 20% of students reported feeling prepared to counsel patients on abortion options [Figure 2]. This low rate was similar for students who completed the didactic ERMD course only and those who completed both the ERMD course and the clinical OBGYN clerkship, with rates of 14% and 25%, respectively. Students who had taken OBGYN reported higher preparedness counseling on male (61%) and female (87%) contraception options, compared to students who had only taken the didactic course (50% and 80%, respectively).

Of note, students with self-reported interest in OBGYN residency were much more likely than students interested in other fields to report feeling prepared to counsel patients about abortion. Half of students interested in OBGYN (50%, 6/12) felt prepared to counsel about abortion, compared to 0% (0/22), 12% (3/25), and 14% (2/14) of students interested in Pediatrics, Internal Medicine, and Emergency Medicine, respectively.

We note that students interested in OBGYN were more likely than other students to seek out extracurricular information surrounding abortion topics, with 100% (18/18) of students interested in OBGYN seeking out extracurricular abortion-related material, as compared to 39% (16/41), 38% (16/42), and 44% (12/27) of students interested in Pediatrics, Internal Medicine, and Emergency Medicine respectively.

### 3.5 Preferred modality of instruction

**Figure 3:**
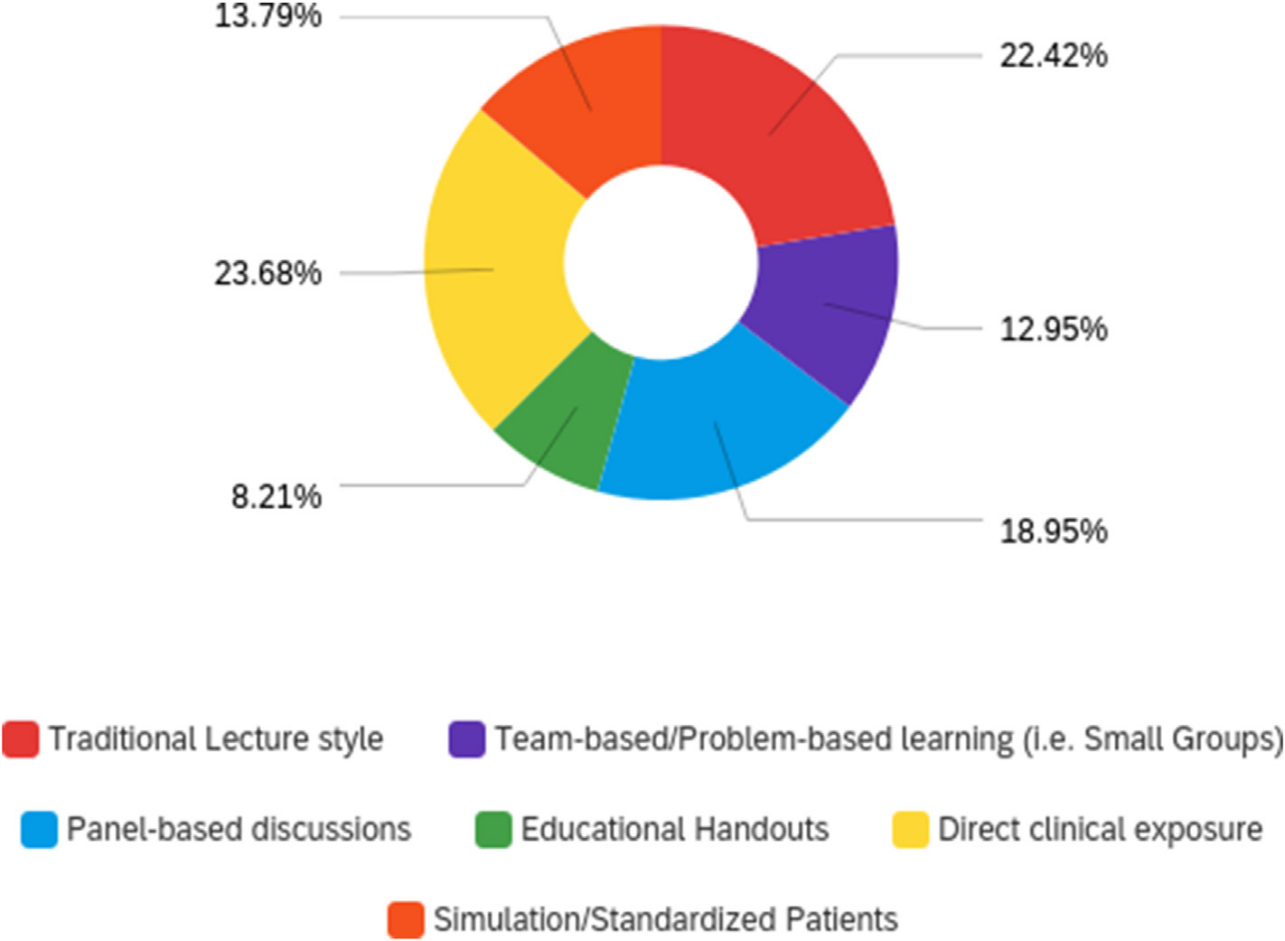
Preferences in modality of instruction on abortion and family planning topics.

Students from all levels of medical education were asked to fill in, “It is best to provide instruction on Family Planning and Contraception via…” and were prompted to select as many answers as they deemed appropriate from the following: traditional lecture style, team-based/problem-based learning (i.e. small group), panel-based discussions, educational handouts, direct clinical exposure, and simulation/standardized patients [Figure 3]. Traditional lecture style (22%) and direct clinical exposure (24%) were the most popular modalities for incorporation of reproductive and sexual health topics into the current curriculum.

## 4 Discussion

We identified discrepancies between the school's SLO and those set by APGO regarding pregnancy termination, but not family planning. We also found a striking disconnect between student interest and feelings of preparedness, exhibiting an important opportunity for intervention.

### 4.1 Interest

The majority of students surveyed expressed interest in topics related to pregnancy termination and family planning. Most third and fourth-year students surveyed who completed the OBGYN clerkship desired more understanding of all options for family planning. Many felt pharmacologic contraception was the only topic thoroughly covered prior to their clerkship, and several mentioned a specific need for awareness of reduced cost options for low-income patients. This reflects a movement within medical education to expand history-taking to include social determinants of health, including poverty, homelessness, and other barriers to access.

Many students cited concerns about how best to discuss contraception and abortion with their patients. Navigating challenging conversations with patients is a competency applicable to multiple specialties. Non-judgmental and empathetic counseling is a necessary skill for all future physicians.

There was minimal education about national or state law surrounding abortion access and the public health implications of abortion-hostile state policy, despite student interest in this topic. While these topics are not necessarily present on national exams such as NBME, USMLE, or COMLEX, making students aware of the impact of reproductive health care policy on patient outcomes and management is a foundational concept not diminished by the rapidly changing nature of the subject matter.

These topics cited by students, including barriers to effective contraceptive use, non-directive pregnancy options counseling, and the public health impact of the legal status of abortion, reflected those found to be absent in the ERMD SLO.

### 4.2 Preparedness to counsel

Most students who completed the ERMD course and/or OBGYN clerkship were not comfortable counseling patients about pregnancy termination. Students interested in OBGYN were more likely than students interested in other specialties to feel prepared to counsel patients about abortion options. Overall, students felt more prepared to counsel patients about their options for non-controversial healthcare interventions, including beta blockers and diuretics. Of note, exposure to clinical experience during the clerkship years did not significantly improve student preparedness to counsel patients about abortion, suggesting that both preclinical and clerkship education are deficient in abortion and reproductive health topics. Consequently, students lack the necessary knowledge and skills to provide routine medical care.

### 4.3 Preferred modality of instruction

Students selected lectures and clinical experience as the most desirable modalities for communicating information about family planning and abortion followed by panel-based discussion, simulated patients, and team-based cases. A study of second- and third-year students at an academic medical center found that clinical exposure to abortion is a valued experience that enabled empathy and challenging of their existing beliefs [8]. While the authors of this paper majorly comment on preclinical exposure to these topics, clinical experience is still a popular educational resource that students value. Clinical experience was associated with more preparedness counseling on male and female contraception options from students who completed their OBGYN clerkship, compared to students who had only taken the didactic course. However, 75% of students who completed their OBGYN clerkship still felt unprepared to counsel on abortion options, indicating that clinical curricula must be addressed and bolstered.

Traditional lecture styles were the second most selected instructional modality. Family planning, particularly contraception counseling, is typically taught as a pharmacology lecture during ERMD. Considering that most students exhibited preparedness in male (61%) and female (87%) contraception counseling, traditional lectures are appropriately covering these topics. However, abortion counseling has not yet been included in a preclinical didactic lecture. Traditional lecturing represents an important entry point for introducing historical and legal knowledge that can enable students to effectively advocate for safe, legal abortions for patients. [9] Compelling and creative abortion and family planning lectures have become more imperative over the last decade. Any lecture on abortion would also necessitate supplying historical, anthropological, and legal frameworks [9].

One avenue for simulated patient exposure is through the objective structured clinical examinations (OSCEs). In a study utilizing a module-based instruction on unplanned pregnancy within a Family Medicine clerkship, 46 third-year students participated [10]. The 10-minute OSCE encounter simulated a woman receiving the diagnosis of an early, unplanned pregnancy. The students were expected to provide nondirective pregnancy options counseling: from delivering news of the pregnancy to presenting options for pregnancy continuation, adoption, and abortion [10]. Afterwards, students reflected on their success in presenting all available options. Such scenario-based learning is a viable option for helping students gain skills and confidence in discussing abortion and contraception.

Panel discussions are an effective method for humanizing illnesses and diseases, as well as exposing medical students to real-life consequences of the social determinants of health, particularly sexual and reproductive health [11]. Exposure to diverse speaker opinions (i.e. practicing medical professionals that do and do not perform abortions) allows students who are opposed to pregnancy termination or uncomfortable with certain reproductive topics to learn how to appropriately treat and counsel patients in ways that reflect their ideologies. While “Panel-based discussion” was the third most popular (19%) instructional modality selected by students, it is an ideal addition to the ERMD course because it offers standardized exposure to course material in a multi-campus medical school and offers more flexible scheduling. Additionally, panels composed of patients and/or providers are often incorporated into relevant courses to stimulate thoughtful conversations around LGBTQ + inclusion, social determinants of health, and healthcare policy, among others. We would likely pursue a panel-based didactic approach over the traditional lecture modality due to the medical school regional campus design, where students are spread geographically across nine campuses in the state of Indiana. Due to the larger concentration of family planning specialists at the urban academic center in Indianapolis with relatively few or no specialists at the other eight regional campuses, we felt that the greatest benefit to all students would be served by a statewide panel where all regional students could have face time with the panelists and have their questions properly answered.

### 4.4 Curriculum integration at other medical institutions

Although techniques, methods and complications of abortion are to be covered in clerkship settings, nondirective counseling, and public health impact of the legal status of abortion are topics that can and should be integrated into didactic courses. Several universities have accomplished this by using Team-Based Learning/Problem-Based Learning (TBL/PBL) forums to encourage open, non-judgmental discussions among students. Two studies conducted in 2013 and 2017 at Northwestern University's Feinberg School of Medicine and the University of Louisville School of Medicine note the successful incorporation of a TBL/PBL about abortion and other pregnancy care into medical school curricula. In the PBL session piloted at the University of Louisville, a pregnant patient diagnosed with Zika virus presented seeking an abortion; the students acted as the providers [12]. The learning objectives for this case reflected the APGO guidelines, and overall, students appreciated the opportunity to discuss pregnancy options counseling and clarify their own values surrounding abortion provision.

At Northwestern University, TBL was used to teach multiple family planning concepts, such as selecting methods of contraception, counseling a patient presenting for a first-trimester abortion, and discussing sterilization on patient request [13]. Compared to the lecture-only study group, the TBL study group reported that the TBL format helped them learn the course material and improve their problem-solving skills (p = .01). Thus, TBL/PBL can be a successful format for introducing pregnancy options counseling and contraception to students in didactic classes [13]. In ERMD and other courses, TBL/PBL is a common pedagogical approach for teaching preclinical themes which necessitate more contextualized discussions, including TBL/PBL on pre-exposure prophylaxis for HIV, organ donation and transplantation, and disability in medicine.

Integration of this content necessitates consideration of the sheer volume of coursework that medical students are expected to learn. Although the amount of medical school content is vast already, we believe that pregnancy options counseling should be introduced in addition to, and not at the loss of, other required content. Pregnancy options counseling–as required in medical student education by APGO–should be incorporated and threaded throughout the medical courses from which students are required to learn. Just as medical students learn briefly about beta blockers in a single pharmacology lecture and then are continuously reminded of their mechanism of action and uses throughout every other course where the drugs have an application, so too should be the logical incorporation of pregnancy options discussions. After all, every single organ system evaluated and taught in medical school is present in pregnant women. In addition to individual TBL/PBL or panel sessions, it is important to thread topics of pregnancy options through cardiovascular, renal, neurological, and musculoskeletal lectures (e.g., “How would you discuss the risks of pregnancy and her options for continuation or termination with a woman who has a congenital heart condition?”) and are reasonable ways to make this content accessible to medical students without overburdening the curriculum.

## 5 Conclusion

Our data is limited by the number and population of students sampled. We received responses from 303 students, representing 21% of the total student body, 36% of whom completed the clerkship and the ERMD course while the remaining students represent the target population for preclinical curricular integration. Additionally, our survey oversampled white students, females, and Midwest natives when compared to the demographic make-up of the school. Response rate was dependent on which students were attentive to the social media outlets we utilized (i.e., GroupMe and Facebook) and the campus newsletter. In future studies, we would aim to sample a larger, more representative proportion of the student body.

Based on our research, we found opportunities for improving the family planning and abortion curriculum. Currently, the ERMD course does not fulfill national APGO learning objectives and fails to prepare students for counseling patients about their options for full-spectrum reproductive health care, including pregnancy options counseling and abortion. Expanding the curriculum to cover family planning and abortion topics is an achievable goal that, based on our survey, would be welcomed by the overwhelming majority of students. Including these topics in preclinical medical curriculum would better prepare students to provide comprehensive, essential, non-judgmental care to patients. While our goal is to provide information to help improve our institution's curriculum, we recognize that there are barriers to incorporating these topics, such as alienating students with differing viewpoints. We hope that by demonstrating student interest and significant knowledge gaps, we have presented sufficient reason to augment the curriculum.

## Declarations

### Author contribution statement

Lucy Brown, BS, BA; Sarah Swiezy, BS; Alexandra McKinzie, BS; Sarah Komanapalli, BS; Caitlin Bernard, MD, MSCI: Conceived and designed the experiments; Performed the experiments; Analyzed and interpreted the data; Contributed reagents, materials, analysis tools or data; Wrote the paper.

### Funding statement

This research did not receive any specific grant from funding agencies in the public, commercial, or not-for-profit sectors.

### Data availability statement

Data will be made available on request.

### Declaration of interests statement

The authors declare no conflict of interest.

### Additional information

No additional information is available for this paper.

## Appendix A Supplementary data

Qualtrics Survey_MSFC Qualtrics Survey_MSFC
