# Supplementary material for: Evaluation of family planning and abortion education in preclinical curriculum at a large midwestern medical school
Source: Heliyon. 2022 Jul 9;8(7):e09894. doi: 10.1016/j.heliyon.2022.e09894 (PMC9283882; doi:10.1016/j.heliyon.2022.e09894)
Supplement: Qualtrics Survey_MSFC [file mmc1.pdf]

Default Question Block

Q1. Dear Respondent,

We are a group of IUSM students conducting a survey to understand IUSM student interest in a comprehensive family planning and contraception curriculum as well as IUSM student perceptions of these topics in the current curriculum. We hope to share the results of this research study with IUSM faculty and the community at large, and to use the results to make changes to guide the curriculum based on IUSM student feedback from this survey.

Your participation in this survey is completely voluntary and is not tied to your grade in any class or clerkship. You will not be penalized for not taking this survey, and you will not be penalized for your responses on this survey if you do take it. There is no way for us to match individual responses on this survey with individuals because we will not be collecting your name, email, or student ID number as a part of this survey. So, please be honest in your responses. You will never be contacted by the survey authors or your course/clerkship instructors about answers provided on this survey.

At the conclusion of this survey, you will be prompted to click a new link where you will enter your email address to be entered into a drawing for one of 6 $25 gift cards for your completion of this survey. This link will be separate from the Qualtrics survey, and there will be no way to match email addresses entered on this separate form to individual survey respondents.

Please reach out to the following emails with questions or concerns about this survey: lucybrow@iu.edu; skomanap@iu.edu; almckinz@iu.edu; sswiezy@iu.edu.

All of the authors of this survey are affiliated with the IUSM chapter of Medical Students For Choice (MSFC). MSFC has provided the funds for the gift cards described above.

Your participation is greatly appreciated!
Sincerely,
Lucy Brown, Sarah Komanapalli, Alexandra McKinzie, and Sarah Swiezy

- ◯ I acknowledge that I have read and understand the above information, and I consent to the use of my responses to this survey as research

Q2. Please indicate which of the following courses you have completed.

- ◯ Endocrine, Reproductive, Musculoskeletal, and Dermatologic Systems (ERMD) UDOS Course AND OB/GYN Clerkship
- ◯ ONLY the Reproductive Block of ERMD UDOS Course
- ◯ Neither

Q3. Please indicate if you identify as:

- ◯ Male
- ◯ Female
- ◯ Nonbinary
- ◯ Transgender
- ◯ Other
- ◯ Prefer not to answer

Q4. Please indicate if you identify as:

- ◯ Gay
- ◯ Lesbian
- ◯ Bisexual

- ◯ Straight
- ◯ Queer
- ◯ Asexual
- ◯ Other
- ◯ Prefer not to say

Q5. Please indicate your race:

- ☐ American Indian or Alaska Native
- ☐ Asian
- ☐ Black or African American
- ☐ Native Hawaiian or Other Pacific Islander
- ☐ White
- ☐ Other
- ☐ Prefer not to answer

Q6. Please indicate your ethnicity

- ◯ Hispanic or Latino
- ◯ Not Hispanic or Latino
- ◯ Prefer not to answer

Q7. What specialty are you most interested in?

- ◯ Undecided
- ◯ Anesthesiology
- ◯ Emergency Medicine
- ◯ Family Medicine
- ◯ Internal Medicine
- ◯ Neurology

- ◯ Obstetrics and Gynecology
- ◯ Pediatrics
- ◯ Psychiatry
- ◯ Radiology
- ◯ Surgery
- ◯ Urology
- ◯ Other

Q8. What is your home geographical region?

- ◯ Northeast
- ◯ Southeast
- ◯ Midwest
- ◯ Southwest
- ◯ West
- ◯ Other

Q9.

Would you be **interested in** including the following topics in Phase I curriculum?

	Yes	No
Abortion counseling	◯	◯
Location of family planning services (Planned Parenthood, OB/GYN, etc.)	◯	◯
Consent, waiting periods, and abortion laws in your state	◯	◯

	Yes	No
Funding for low-income women seeking family planning	◯	◯
Pharmacological methods of contraception	◯	◯
Surgical and non-surgical methods of abortion	◯	◯

Q10.

Do you feel that these reproductive topics were **adequately covered** in your Phase I curriculum?

	Yes	No
Abortion counseling	◯	◯
Location of family planning services (Planned Parenthood, OB/GYN, etc.)	◯	◯
Consent, waiting periods, and abortion laws in your state	◯	◯
Funding for low-income women seeking family planning	◯	◯
Pharmacological methods of contraception	◯	◯
Surgical and non-surgical methods of abortion	◯	◯

Q11.
Have you sought out extracurricular experiences to enhance your understanding of the topics listed above?

- ◯ Yes
- ◯ No

Q12. Where did you seek this extra information?

- ☐ USMLE Resources (eg. First Aid, Boards and Beyond, Pathoma)
- ☐ IUSM Student Organizations (eg. OB/GYN SIG, MSFC)
- ☐ External Organizations (eg. Planned Parenthood)
- ☐ Other

Q13. Do you feel **PREPARED** to counsel patients on the following?

	Yes	No
Beta blockers	◯	◯
Abortion options	◯	◯
Male Contraception options	◯	◯
Female Contraception options	◯	◯
Anti-depressant/Anti-anxiety treatment and therapies	◯	◯
Diuretics	◯	◯

Q14. It is best to provide instruction on **Family Planning and Contraception** in...

NOTE: Please select as many answers as you deem appropriate

- ☐ Medical School, pre-clinical years
- ☐ Medical School, clinical years
- ☐ Residency
- ☐ Post-Graduate Continuing Medical Education (i.e. Fellowship)
- ☐ Not necessary to provide formalized instruction on Family Planning and Contraception

Q15. It is best to provide instruction on **Family Planning and Contraception** via...

NOTE: Please select as many answers as you deem appropriate

- ☐ Traditional Lecture style
- ☐ Team-based/Problem-based learning (i.e. Small Groups)
- ☐ Panel-based discussions
- ☐ Educational Handouts
- ☐ Direct clinical exposure
- ☐ Simulation/Standardized Patients

Q16. Indicate how strongly you agree/disagree with the following statement:

Regardless of personal views about abortion, students should be knowledgeable about its public health importance as well as techniques and complications.

- ○ Strongly agree
- ○ Agree
- ○ Somewhat agree
- ○ Somewhat disagree

- ◯ Disagree
- ◯ Strongly disagree

Q17. This is a standardized question. Please choose the color "yellow" from the list below.

- ◯ Blue
- ◯ Yellow
- ◯ Red
- ◯ Green

Q18.
How effectively do you think the current IUSM curriculum covers sexual and reproductive health topics, including contraception and family planning?

- ◯ Very effectively
- ◯ Somewhat effectively
- ◯ Somewhat ineffectively
- ◯ Very ineffectively

Q19. How well do you feel that Phase I didactic courses prepared you for the family planning and contraception component of the OB/GYN Clerkship?

- ◯ Extremely well
- ◯ Very well
- ◯ Slightly well
- ◯ Not well at all

Q20. What do you wish you had known about family planning and contraception before starting your OB/GYN Clerkship?

Please answer openly and truthfully.

Q21.
Do you believe that IUSM should enhance its reproductive and sexual health coverage in the current curriculum, including expanding family planning and contraception didactic training?

- ◯ Yes
- ◯ No

Q22.
In which courses do reproductive health topics belong?

- ☐ Human Structure
- ☐ Molecules to Cells to Tissues
- ☐ Fundamentals of Health and Disease
- ☐ Host Defense
- ☐ Neuroscience and Behavior
- ☐ Cardiovascular and Hematology
- ☐ Renal and Respiratory
- ☐ Gastrointestinal and Nutrition
- ☐ Endocrine, Reproductive, Musculoskeletal and Dermatologic
- ☐ Foundations of Clinical Practice I
- ☐ Foundations of Clinical Practice II
- ☐ All of the above

Q23. This link will re-direct you to a google form where you can enter your IU email address (please only submit one address) for a drawing for a $25 gift

card. Your survey responses cannot be linked to the email address you provide on the Google Form.

Please SUBMIT your survey before proceeding to the Google Form.

https://forms.gle/VdTdbkRFzYQVuavN8

Powered by Qualtrics
